# Editorial: Role of HLA and KIR in Viral Infections

**DOI:** 10.3389/fimmu.2016.00286

**Published:** 2016-07-27

**Authors:** Jelle de Wit, José A. M. Borghans, Can Kesmir, Debbie van Baarle

**Affiliations:** ^1^Department of Immune Mechanisms, Centre for Infectious Disease Control, National Institute for Public Health and the Environment, Bilthoven, Netherlands; ^2^Laboratory of Translational Immunology, Department of Immunology, University Medical Center, Utrecht, Netherlands; ^3^Theoretical Biology and Bioinformatics, Utrecht University, Utrecht, Netherlands

**Keywords:** killer Ig-like receptors, human leukocyte antigen, T cell receptor, natural killer cells, antiviral immune responses

**The Editorial on the Research Topic**

**Role of HLA and KIR in Viral Infections**

The immune system continuously protects its host against pathogens. During viral infections, both innate and adaptive immune cells contribute to an effective immune response. Natural killer (NK) cells can respond quickly to eliminate pathogens and infected cells and suppress dissemination to other tissues. Subsequently, activation of virus-specific CD8^+^ cytotoxic T cells results in specific killing of infected cells and activation of virus-specific CD4^+^ T cells further supports the immune response. Human leukocyte antigens (HLA) play an essential role in activation of both NK cells and T cells. This research topic contains eight articles highlighting the latest insights into the various effects of HLA molecules on both NK cell and T-cell reactivity upon viral infection.

Natural Killer cell activation is regulated by a variety of activating and inhibitory receptors, including killer-cell immunoglobulin-like receptors (KIRs). KIRs bind to HLA class I molecules, which are expressed on all nucleated cells. As HLA and KIR molecules are highly polymorphic, each individual expresses a unique set of these molecules. The wide range of combinations of HLA and KIR expression results in differences in binding strengths and variation in NK cell activation (1). In this research topic, a review by Walter and Ansari illuminates the associations of HLA and KIR polymorphisms with the outcome of experimental simian immunodeficiency virus infection in rhesus macaques, a model used to study human immunodeficiency virus (HIV) infection. Not only do these associations show which interactions contribute to disease resistance, they also pinpoint combinations that increase susceptibility to disease. Such associations of HLA and KIR with disease progression have also been found in hepatitis C virus (HCV) infection. In her review, Gardiner summarizes current insights into these associations, and further illustrates how NK cells modulate disease outcome in HCV infection. Furthermore, this review highlights the ongoing search for the NK cell subsets required for protective host responses.

In addition to KIRs, NK cells express C-type lectin receptors, including NKG2C, which regulate activation *via* ligation to non-classical HLA-E molecules. Della Chiesa et al. summarize the role of activating KIRs and NKG2C in several virus infections, including human cytomegalovirus (HCMV) and HIV. In addition, they discuss the induction of memory-like NK cells, which show enhanced responses upon reinfection and may play a role in controlling recurrent or chronic infections. Expression of NKG2C has also been associated with expansion of NK cells during viral infection. This expansion of NKG2C^+^ NK cells with expression of self-HLA class I-specific KIRs has been observed in HCMV infection (2). The force driving these NK cell expansions is largely unclear. In this collection, Beziat et al. investigate the importance of HLA class I expression levels in host defense using data derived from transporter associated with antigen processing (TAP)-deficient individuals who express less than 10% of normal HLA class I levels. They demonstrate that self-HLA class I molecules shape the KIR repertoire of NKG2C^+^ NK cells, but are not a requirement for expansion.

Besides influencing innate NK responses *via* ligation to KIRs, HLA induces adaptive immune responses by presenting pathogen-derived peptides to T cells. Recognition of HLA:peptide complexes by a peptide-specific T-cell receptor leads to activation of specific CD8^+^ cytotoxic T cells or CD4^+^ T helper cells. In HCMV infection, effective CD8^+^ T-cell responses have been shown to be dominated by peptides derived from immediate-early 1 (IE-1) protein (3–5). Whether IE-1 is also the dominant peptide source for CMV-specific CD4^+^ T cell responses is unclear. Using cells derived from healthy HCMV-positive donors, Ameres et al. generated multiple IE-1-specific CD4^+^ T cell clones with a highly diverse repertoire and found that IE-1-specific CD4^+^ T cells participate in the antiviral response. Since both CD4^+^ and CD8^+^ T cells respond to IE-1-derived peptides, it might be an interesting target for immunotherapeutic approaches.

Infection with measles virus is known to induce a strong T cell response (6, 7), but information regarding the specific measles virus antigens that are responsible for activation of these cells is limited. Schellens et al. investigated which measles peptides are presented by HLA class I molecules by eluting naturally presented peptides from virus-infected cells. They show that a broad spectrum of the measles peptidome is presented by different HLA class I molecules. Furthermore, they found that while HLA-B molecules present the most diverse set of peptides, the abundant epitopes were eluted from HLA-A and HLA-C molecules, suggesting that the HLA loci also influence peptide presentation.

Due to the polymorphic nature of HLA molecules, a great diversity of peptides are typically presented, resulting in T-cell activation of variable strengths. In HCV infections, some HLA molecules (e.g., HLA-B27 and HLA-B57) are significantly associated with viral clearance (8, 9). However, it is not yet known why such HLA molecules provide an advantage during HCV infections. Using known HCV epitopes combined with *in silico* predictions, Rao et al. demonstrate that HLA-B27 preferentially presents epitopes from the HCV protein NS5B, which is highly conserved and, therefore, might be one of the underlying mechanisms behind the protective effect of this molecule during HCV infection. Preferential presentation of peptides derived from conserved regions was also found for HIV (10–12). However, the induction of effective immune responses also puts pressure on the virus to adapt toward escape variants. The generation of escape variants often comes with significant costs, such as reduced viral replicative capacity. In the final paper of this issue, a review by KlØverpris et al. illustrates the effects of viral escape variants within the HIV-infected patient as well as the consequences of transmission of these variants at the population level.

Together, the research and reviews in this research topic provide an up-to-date overview of the importance of HLA and KIR molecules in host responses to viral infection. Given the central role of HLA in modulating both innate and adaptive immune responses, it is important to expand our knowledge on the role of different HLA molecules in various diseases. A better understanding of the differences between the individual immune responses, especially when it fails to protect against pathogens, should ultimately help to develop better – and possibly more individualized – treatment options.

## Author Contributions

JW, JB, CK, and DB wrote the editorial.

## Conflict of Interest Statement

The authors declare that the research was conducted in the absence of any commercial or financial relationships that could be construed as a potential conflict of interest.
